# Patient and physician global assessments capture distinct clinical domains in axial spondyloarthritis

**DOI:** 10.1007/s10067-026-08254-0

**Published:** 2026-06-25

**Authors:** Duygu Kurtulus, Ekrem Aslan, Adem Kucuk

**Affiliations:** 1 Department of Physical Medicine and Rehabilitation, Faculty of Medicine, Uskudar University,Memorial Atasehir Hospital, Istanbul, Turkey; 2 Department of Internal Medicine, Division of Gastroenterology, Memorial Atasehir Hospital, Istanbul, Turkey; 3 Department of Internal Medicine, Division of Rheumatology, Faculty of Medicine, Necmettin Erbakan University, Konya, Turkey

**Keywords:** Axial spondyloarthritis, Depression, Disease activity, Fatigue, Inflammation, Patient global assessment, Physician global assessment, Spinal mobility

## Abstract

**Background:**

Patient global (PtGA) and physician global (PhGA) assessments are key components of disease evaluation in axial spondyloarthritis (axSpA). Although commonly used in composite indices, these measures are often interpreted as interchangeable indicators of disease activity. It remains unclear whether PtGA and PhGA reflect overlapping or distinct clinical domains.

**Methods:**

In this cross-sectional study, 155 patients fulfilling the modified New York criteria, with ASAS classification criteria additionally recorded, were included. All patients had radiographic and MRI-confirmed sacroiliitis. Patient-reported outcomes included PtGA (0–10 VAS)**,** fatigue (0–100), and Beck Depression Inventory (BDI). PhGA (0–10 VAS) was assessed independently by the evaluating physician. Laboratory parameters included erythrocyte sedimentation rate (ESR) and C-reactive protein (CRP). Spinal mobility was evaluated using the modified Schober test, tragus-to-wall distance, and chest expansion. Associations were examined using Spearman correlation and multivariate linear regression analyses including demographic variables.

**Results:**

PtGA showed strong correlations with depressive symptoms (*ρ* = 0.61, *p* < 0.001) and fatigue (*ρ* = 0.53, *p* < 0.001), while correlations with inflammatory markers were weak. In multivariate analyses, BDI (*β* = 0.087, *p* < 0.001), fatigue (*β* = 0.015, *p* = 0.002), ESR (*β* = 0.040, *p* = 0.001), disease duration (*β* =  − 0.068, *p* = 0.036), and tragus-to-wall distance (*β* = 0.069, *p* = 0.040) remained independently associated with PtGA (adjusted *R*^2^ = 0.402), whereas spinal mobility measures were not significant. In contrast, PhGA was independently associated with modified Schober (*β* =  − 0.200, *p* = 0.031), tragus-to-wall distance (*β* = 0.061, *p* = 0.043), ESR (*β* = 0.031, *p* = 0.004), and BDI (*β* = 0.075, *p* < 0.001) (adjusted *R*^2^ = 0.434). CRP and demographic variables were not significant predictors of either global assessment.

**Conclusion:**

Patient and physician global assessments capture distinct clinical domains in axSpA. PtGA is predominantly influenced by psychological burden and fatigue, whereas PhGA is more strongly associated with structural impairment and inflammatory burden. These findings highlight the multidimensional nature of disease perception and support domain-targeted clinical evaluation.

**Table:** 

**Key Points** • *Patient and physician global assessments in axial spondyloarthritis reflect partially overlapping but distinct clinical domains.* • *Patient global assessment is primarily driven by depressive symptoms and fatigue rather than objective inflammatory or structural parameters.* • *Physician global assessment is independently associated with spinal mobility impairment and inflammatory burden.* • *Interpreting PG–MD differences as domain-specific weighting rather than discordance may improve multidimensional disease assessment.*

## Introduction

Global assessments are central components of disease evaluation in axial spondyloarthritis (axSpA) and are incorporated into composite indices used in both clinical practice and research [1–3]. Patient global assessment (PtGA) reflects the patient’s overall perception of disease activity, whereas physician global assessment (PhGA) represents the clinician’s evaluation of inflammatory and functional status [2, 4]. These measures are typically obtained using numerical rating scales or visual analog scales and are not specific to individual disease domains. Although both measures are widely used, they are often treated as interchangeable indicators of disease burden.

Previous studies have highlighted discrepancies between patient and physician global assessments, frequently framing these differences as patient–physician discordance [5–7]. However, limited attention has been paid to the possibility that PtGA and PhGA may inherently reflect distinct clinical domains rather than measurement disagreement. In axial spondyloarthritis, disease expression is multidimensional, encompassing inflammatory activity, structural damage, functional limitation, fatigue, and psychological burden [1, 8, 9]. It remains unclear to what extent these components differentially influence patient and physician perceptions of disease severity.

Understanding the determinants of global assessments is clinically relevant. If PtGA is primarily driven by subjective symptom burden and psychological factors, while PhGA is more closely linked to objective structural or inflammatory parameters, interpreting discordance solely as misalignment may be misleading [6, 7]. Clarifying the domain-specific drivers of PtGA and PhGA may therefore contribute to more nuanced clinical decision-making and individualized management strategies.

The present study aimed to evaluate the clinical, psychological, inflammatory, and functional determinants of patient and physician global assessments in a well-characterized cohort of patients with radiographic axial spondyloarthritis. By examining these associations through multivariate modeling, we sought to determine whether PtGA and PhGA capture overlapping or distinct dimensions of disease expression.

## Materials and methods

This cross-sectional observational study was conducted between August 2025 and October 2025 and included 155 prospectively recruited from the outpatient clinic patients fulfilling the modified New York criteria [10]. In addition, the Assessment of SpondyloArthritis International Society (ASAS) classification criteria for axial spondyloarthritis [1] were also recorded to ensure compatibility with contemporary classification systems.

All patients had available radiographic and MRI sacroiliitis, ensuring objective structural evidence of axial involvement. Radiographic sacroiliitis was defined according to the modified New York criteria (bilateral grade ≥ 2 or unilateral grade ≥ 3). MRI findings were evaluated for active inflammatory lesions. Imaging modalities were not necessarily performed simultaneously but reflected documented evidence of structural and/or inflammatory involvement during the disease course. Quantitative MRI scoring systems were not applied, as the present analysis focused on clinical, functional, and laboratory determinants of patient and physician global assessments.

Eligible participants were ≥ 18 years of age and had complete clinical and functional data at the time of assessment. This was a cross-sectional study with prospective data collection, and all variables were obtained at a single study visit using standardized forms. No retrospective data extraction was performed. Laboratory parameters were available for a subset of patients and were handled using a complete-case approach in regression analyses. Exclusion criteria included diagnosed psychiatric or neurological disorders, malignancy, fibromyalgia, chronic widespread pain syndrome, mechanical low back pain unrelated to axial spondyloarthritis, and other inflammatory rheumatic diseases in order to minimize potential confounding effects on patient-reported outcomes.

Demographic data (age, sex, and disease duration), HLA-B27 status, and comorbidities were recorded. Comorbidities were obtained through patient history and review of medical records. Patient-reported outcomes included the patient global assessment (PtGA, 0–10 cm visual analog scale), fatigue score (numeric rating scale, 0–100), and the Beck Depression Inventory (BDI). The physician global assessment (PhGA, 0–10 cm visual analog scale) was completed independently by the evaluating physician, who was blinded to PtGA and fatigue scores.

Laboratory parameters included erythrocyte sedimentation rate (ESR) and C-reactive protein (CRP), measured using standard laboratory techniques. Laboratory measurements were obtained within the same clinical visit or within a maximum interval of 1 week.

Spinal mobility was assessed using the modified Schober test, chest expansion, tragus-to-wall distance, and chin-to-sternum distance. All physical examinations and measurements were performed by the same physician to ensure consistency and reduce inter-observer variability.

Missing data were minimized through prospective data collection using structured assessments. No imputation methods were applied, and analyses were conducted using complete-case data.

The study protocol was approved by the Ethics Committee of the University of Health Sciences, Ümraniye Training and Research Hospital (approval number: B.10.1.TKH.4.34.HGP.0.01/234). Although patient recruitment was not conducted at this institution, ethical approval was obtained in accordance with national regulations. Written informed consent was obtained from all participants prior to enrollment. The study was conducted in accordance with the principles of the Declaration of Helsinki (2024 revision) and adhered to the STROBE guidelines for cross-sectional studies.

### Statistical analysis

All statistical analyses were performed using IBM SPSS Statistics for Windows, version 25.0 (IBM Corp., Armonk, NY, USA). The normality of continuous variables was assessed using the Shapiro–Wilk test and visual inspection of histograms. As most variables were not normally distributed, continuous data are presented as median (interquartile range [IQR]), while categorical variables are expressed as frequencies and percentages.

Associations between patient global assessment (PtGA), physician global assessment (PhGA), psychological measures, inflammatory markers, and spinal mobility parameters were evaluated using Spearman’s rank correlation coefficient (*ρ*).

To identify independent determinants of PtGA and PhGA, multivariate linear regression analyses were performed using a complete-case approach (listwise deletion of missing data). Two regression models were constructed to account for incomplete laboratory data. In the first model, age, sex, disease duration, psychological variables (Beck Depression Inventory and fatigue score), and spinal mobility measures (modified Schober test and tragus-to-wall distance) were entered simultaneously. In the second model, inflammatory markers (erythrocyte sedimentation rate and C-reactive protein) were additionally included.

Regression coefficients (*β*), corresponding *p* values, and adjusted *R*^2^ values are reported. Multicollinearity was assessed using variance inflation factor (VIF) statistics; all VIF values were below 2.5, indicating no significant collinearity among predictors. Model assumptions were evaluated through inspection of residual plots and were considered adequately met. A two-tailed *p* value < 0.05 was considered statistically significant.

## Results

A total of 155 patients fulfilling the modified New York with ASAS classification criteria additionally recorded were included in the study. All patients had available radiographic and MRI evidence of sacroiliitis**.** Baseline demographic, clinical, laboratory, and spinal mobility characteristics are presented in Table 1.

**Table 1:** Baseline clinical and demographic characteristics (*n* = 155)

Variable	Median (IQR)/*n* (%)
Age (years)	34.0 (28.5–40.0)
Female sex, *n* (%)	48 (31.0%)
Disease duration (years)	19.0 (16.0–25.5)
HLA-B27 positivity, *n* (%)	86 (55.5%)
Patient global (VAS 0–10)	5.0 (3.5–7.0)
Physician global (VAS 0–10)	5.0 (3.0–7.0)
Beck Depression Inventory	16.0 (9.0–23.75)
Fatigue (0–100)	30.0 (5.75–60.0)
ESR (mm/h)	12.5 (6.0–20.75)
CRP (mg/dL)	0.85 (0.32–1.88)
Modified Schober (cm)	4.0 (2.0–5.5)
Tragus-to-wall (cm)	19.0 (16.0–23.0)
Chest expansion (cm)	3.5 (2.0–5.0)

Continuous variables are presented as median (interquartile range [IQR]) due to non-normal distribution assessed by the Shapiro–Wilk test

Spearman correlation coefficients between PtGA**,** PhGA, psychological variables, inflammatory markers, and spinal mobility parameters are summarized in Table 2.

**Table 2:** Spearman correlations with global assessments

Variable	PtGA (*ρ*)	PhGA (*ρ*)
Beck Depression Inventory	0.61***	0.50***
Fatigue	0.53***	0.53***
ESR	0.18*	0.26**
CRP	0.19*	0.31**
Modified Schober	− 0.20*	− 0.43***
Tragus-to-wall	0.22*	0.38***
Chest expansion	0.04	− 0.23*

Spearman’s rank correlation coefficients (*ρ*) are presented. *p < 0.05, **p < 0.01, ***p < 0.001. Continuous variables were non-normally distributed (Shapiro–Wilk) and were analyzed using Spearman correlation

Spearman correlation analysis demonstrated distinct association patterns for PtGA and PhGA assessments. PtGA showed strong positive correlations with Beck Depression Inventory (*ρ* = 0.61, *p* < 0.001) and fatigue (*ρ* = 0.53, *p* < 0.001), whereas correlations with inflammatory markers were weak (ESR ρ = 0.18, *p* = 0.041**;** CRP ρ = 0.19, *p* = 0.036). Associations between PtGA and spinal mobility parameters were modest (modified Schober *ρ* =  − 0.20, *p* = 0.018; tragus-to-wall distance *ρ* = 0.22, *p* = 0.012), and no meaningful correlation was observed with chest expansion (ρ = 0.04, *p* = 0.62).

PhGA demonstrated significant correlations with both psychological and structural parameters. Strong correlations were observed with spinal mobility measures, including modified Schober (*ρ* =  − 0.43, *p* < 0.001) and tragus-to-wall distance (*ρ* = 0.38, *p* < 0.001). PhGA also correlated with BDI (*ρ* = 0.50, *p* < 0.001) and fatigue (*ρ* = 0.53, *p* < 0.001), as well as inflammatory markers (ESR *ρ* = 0.26; CRP *ρ* = 0.31).

To determine independent predictors, multivariate regression analyses were performed.

Model 1, demographic variables (age, sex, disease duration), including psychological and mobility variables, was conducted in 141 patients with complete data (Table 3). In this model, BDI (*β* = 0.082, *p* < 0.00) and fatigue (*β* = 0.022, *p* < 0.001) and di sease duration (*β* =  − 0.080, *p* = 0.011) remained independently associated with PtGA. Age (*p* = 0.994) and sex (*p* = 0.722) were not significant predictors. Spinal mobility parameters were not significant predictors. The model explained approximately 41% of the variance (adjusted *R*^2^ = 0.406). For MD, modified Schober and tragus-to-wall distance remained independently associated, while fatigue did not retain statistical significance.

**Table 3:** Multivariate linear regression analysis identifying determinants of PtGA

Variable	Model 1 (*n* = 141) *β* (95% CI), *p* value	Model 2 (*n* = 120) *β* (95% CI), *p* value
Age	− 0.000 (− 0.038 to 0.038), *p* = 0.994	− 0.005 (− 0.045 to 0.035), *p* = 0.791
Sex	− 0.142 (− 0.931 to 0.646), *p* = 0.722	− 0.112 (− 0.964 to 0.739), *p* = 0.794
Disease duration	− 0.080 (− 0.141 to − 0.018), *p* = 0.011*	− 0.068 (− 0.131 to − 0.004), *p* = 0.036*
Beck Depression Inventory	0.082 (0.048 to 0.116), *p* < 0.001*	0.087 (0.051 to 0.123), *p* < 0.001*
Fatigue	0.022 (0.013 to 0.030), *p* < 0.001*	0.015 (0.006 to 0.025), *p* = 0.002*
Modified Schober	− 0.149 (− 0.328 to 0.030), *p* = 0.102	− 0.074 (− 0.272 to 0.125), *p* = 0.462
Tragus-to-wall	0.050 (− 0.013 to 0.112), *p* = 0.121	0.069 (0.003 to 0.135), *p* = 0.040*
ESR	—	0.040 (0.017 to 0.064), *p* = 0.001*
CRP	—	− 0.027 (− 0.065 to 0.010), *p* = 0.155
Adjusted R^2^	0.406	0.402

Multivariate linear regression analyses were performed using complete-case data. Model 1 included demographic variables (age, sex, disease duration), psychological variables, and spinal mobility measures. Model 2 additionally included inflammatory markers (ESR and CRP).* β* values represent unstandardized regression coefficients with 95% confidence intervals

*Statistically significant variables

For PhGA, modified Schober and tragus-to-wall distance remained independently associated in Model 1, whereas fatigue did not retain statistical significance.

Model 2 additionally incorporated inflammatory markers (ESR and CRP) and was conducted in 120 patients with complete laboratory data (Table 4). In the fully adjusted model for PtGA, BDI (*β* = 0.087, *p* < 0.001), fatigue (*β* = 0.015, *p* = 0.002), and ESR (β = 0.040, *p* = 0.001), disease duration (*β* =  − 0.068, *p* = 0.036), and tragus-to-wall distance (*β* = 0.069, *p* = 0.040) remained independently associated with PtGA. Age (*p* = 0.791) and sex (*p* = 0.794) remained non-significant, and CRP (*p* = 0.155) was not independently associated. The model explained approximately 40% of the variance (adjusted *R*^2^ = 0.402).

**Table 4:** Multivariate linear regression analysis ıdentifying determinants of PhGA

Variable	Model 1 (*n* = 141) *β* (95% CI), *p* value	Model 2 (*n* = 120) *β* (95% CI), *p* value
Age	0.016 (− 0.021 to 0.053), *p* = 0.393	0.009 (− 0.030 to 0.048), *p* = 0.647
Sex	− 0.316 (− 1.076 to 0.444), *p* = 0.410	− 0.120 (− 0.937 to 0.697), *p* = 0.773
Disease duration	− 0.014 (− 0.076 to 0.048), *p* = 0.659	− 0.010 (− 0.074 to 0.054), *p* = 0.759
Beck Depression Inventory	0.072 (0.039 to 0.105), *p* < 0.001*	0.075 (0.040 to 0.110), *p* < 0.001*
Fatigue	0.010 (0.002 to 0.018), *p* = 0.022*	0.007 (− 0.003 to 0.016), *p* = 0.153
Modified Schober	− 0.312 (− 0.485 to − 0.139), *p* < 0.001*	− 0.200 (− 0.381 to − 0.019), *p* = 0.031*
Tragus-to-wall	0.039 (− 0.019 to 0.097), *p* = 0.182	0.061 (0.002 to 0.121), *p* = 0.043*
ESR	—	0.031 (0.010 to 0.052), *p* = 0.004*
CRP	—	0.003 (− 0.028 to 0.034), *p* = 0.839
Adjusted *R*^2^	0.434	0.434

Multivariate linear regression analyses were performed using complete-case data. Model 1 included demographic variables (age, sex, disease duration), psychological variables, and spinal mobility measures. Model 2 additionally included inflammatory markers (ESR and CRP). *β* values represent unstandardized regression coefficients with 95% confidence intervals

*Statistically significant variables

## Discussion

In this cross-sectional study of patients with radiographic axial spondyloarthritis, we demonstrated that patient and physician global assessments reflect partially overlapping but distinct clinical domains. While both measures were associated with psychological and inflammatory parameters, their independent determinants differed substantially. PtGA was primarily driven by depressive symptoms and fatigue, whereas PhGA assessment was more strongly associated with objective spinal mobility impairment and inflammatory burden.

Our findings indicate that patient global assessment is predominantly influenced by subjective symptom perception. In multivariate analyses, depressive symptoms and fatigue consistently remained independent predictors of PtGA, whereas spinal mobility measures did not. The association between depressive symptoms and higher perceived disease activity is well documented in axial spondyloarthritis [8]. Fatigue has likewise been identified as a major determinant of patient-reported disease burden in axSpA [11]. Moreover, comorbid fibromyalgia and central sensitization mechanisms may further amplify symptom perception independent of objective inflammation [12]. Although ESR showed a modest independent association, CRP did not retain significance in the fully adjusted model. This finding aligns with prior evidence highlighting the limited sensitivity and variability of CRP in axial spondyloarthritis [13]. These results suggest that PtGA reflects the overall illness experience rather than structural disease severity alone. Notably, disease duration also emerged as an independent determinant, suggesting that chronicity contributes to symptom perception beyond current inflammatory activity [14].

In contrast, physician global assessment demonstrated a different weighting pattern. Spinal mobility measures—particularly modified Schober test and tragus-to-wall distance—remained independently associated with PhGA across models. ESR also retained independent significance, while CRP did not. Notably, although PhGA correlated with psychological measures in univariate analysis, fatigue did not remain significant in multivariate models, suggesting that physician assessment is less influenced by subjective symptom amplification and more anchored in objective structural and inflammatory parameters [4, 5, 15–17]. Importantly, demographic variables (age, sex, and disease duration) were not independently associated with PhGA, indicating that clinical evaluation is primarily driven by structural and inflammatory parameters rather than demographic characteristics.

The absence of an independent association between CRP and either global assessment in fully adjusted models is noteworthy. This may reflect the known variability and limited sensitivity of CRP in axial spondyloarthritis, particularly in patients with predominantly structural damage rather than active inflammation [13]. ESR, in contrast, retained independent associations, possibly reflecting its more stable relationship with systemic inflammatory burden in this cohort [13, 16].

Taken together, these findings support the concept that patient and physician global assessments do not measure identical aspects of disease activity [3, 4, 15, 16, 18]. Rather than representing discordance per se, the two measures appear to capture different dimensions of disease expression: PtGA reflecting subjective symptom burden and psychological impact and PhGA reflecting structural impairment and inflammatory status [3, 18]. This distinction has important clinical implications within a treat-to-target framework emphasizing multidimensional disease assessment [1, 18, 19]. Elevated PtGA in the absence of objective mobility restriction or inflammatory activity may signal unmet psychological or fatigue-related needs rather than uncontrolled inflammatory disease [8, 11, 20], whereas elevated PhGA scores may more directly reflect structural progression or inflammatory burden [15, 21].

BASDAI and BASFI were not included in the regression models because these composite indices contain patient-reported symptom and functional domains that conceptually overlap with patient global assessment and spinal mobility measures. Instead, their underlying components (fatigue, psychological burden, and objective mobility parameters) were entered separately to minimize collinearity and to better delineate domain-specific determinants of global assessments.

Our study has several limitations. The cross-sectional design precludes causal inference, and longitudinal studies are needed to determine whether these domain-specific associations predict treatment response or structural progression. In addition, quantitative MRI scoring systems were not applied, which may have further refined the assessment of inflammatory activity. However, all patients had radiographic and MRI-confirmed sacroiliitis, ensuring structural disease homogeneity [3, 20, 22, 23].

Despite these limitations, the study has important strengths, including a well-characterized cohort with objective structural confirmation, standardized physician assessment, and multivariate modeling accounting for psychological, inflammatory, and functional domains. By clarifying the distinct determinants of patient and physician global assessments, our findings contribute to a more nuanced understanding of disease perception in axial spondyloarthritis.

## Conclusion

Patient and physician global assessments in axial spondyloarthritis reflect distinct clinical domains rather than interchangeable measures of disease activity. While PtGA is predominantly influenced by depressive symptoms and fatigue, PhGA is more strongly associated with spinal mobility impairment and inflammatory burden. Demographic variables were not independently associated with either global assessment, reinforcing the predominance of clinical and disease-related factors. These findings highlight the multidimensional nature of disease perception and underscore the importance of integrating both subjective and objective parameters in clinical evaluation. Recognizing the domain-specific determinants of global assessments may facilitate more individualized and domain-targeted management strategies in clinical practice.

## Data Availability

The datasets generated and analyzed during the current study are available from the corresponding author on reasonable request, in accordance with institutional policies and ethical regulations.
